# Effect of a coaching intervention to enhance physical activity and prevent falls in community-dwelling people aged 60+ years: a cluster randomised controlled trial

**DOI:** 10.1136/bjsports-2023-107027

**Published:** 2024-01-22

**Authors:** Juliana S Oliveira, Catherine Sherrington, Chris Rissel, Kirsten Howard, Allison Tong, Dafna Merom, James Wickham, Adrian E Bauman, Stephen R Lord, Richard I Lindley, Judy M Simpson, Margaret Allman-Farinelli, Catherine Kirkham, Elisabeth Ramsay, Sandra O’Rourke, Anne Tiedemann

**Affiliations:** 1 Sydney Musculoskeletal Health, The University of Sydney, Sydney, New South Wales, Australia; 2 Institute for Musculoskeletal Health, The University of Sydney and Sydney Local Health District, Sydney, New South Wales, Australia; 3 Sydney School of Public Health, The University of Sydney, Sydney, New South Wales, Australia; 4 Menzies Centre for Health Policy and Economics, Sydney School of Public Health, The University of Sydney, Sydney, New South Wales, Australia; 5 School of Health Sciences, Western Sydney University, Sydney, New South Wales, Australia; 6 School of Dentistry and Medical Sciences, Charles Sturt University, Orange, New South Wales, Australia; 7 Prevention Research Collaboration, Sydney School of Public Health, The University of Sydney, Sydney, New South Wales, Australia; 8 Neuroscience Research Australia, Randwick, New South Wales, Australia; 9 Sydney Medical School, Discipline of Medicine, The University of Sydney, Sydney, New South Wales, Australia; 10 Nutrition and Dietetics, School of Nursing, Faculty of Medicine and Health, The University of Sydney, Sydney, New South Wales, Australia

**Keywords:** Accidental Falls, Physical activity, Aging

## Abstract

**Objectives:**

To evaluate the effect of a coaching intervention compared with control on physical activity and falls rate at 12 months in community-dwelling people aged 60+ years.

**Design:**

Cluster randomised controlled trial.

**Setting:**

Community-dwelling older people.

**Participants:**

72 clusters (605 participants): 37 clusters (290 participants) randomised to the intervention and 35 (315 participants) to control.

**Intervention:**

Intervention group received written information, fall risk assessment and prevention advice by a physiotherapist, activity tracker and telephone-based coaching from a physiotherapist focused on safe physical activity. Control group received written information and telephone-based dietary coaching. Both groups received up to 19 sessions of telephone coaching over 12 months.

**Outcomes:**

The co-primary outcomes were device-measured physical activity expressed in counts per minute at 12 months and falls rate over 12 months. Secondary outcomes included the proportion of fallers, device-measured daily steps and moderate-to-vigorous physical activity (MVPA), self-reported hours per week of physical activity, body mass index, eating habits, goal attainment, mobility-related confidence, quality of life, fear of falling, risk-taking behaviour, mood, well-being and disability.

**Results:**

The mean age of participants was 74 (SD 8) years, and 70% (n=425) were women. There was no significant effect of the intervention on device-measured physical activity counts per minute (mean difference 5 counts/min/day, 95% CI −21 to 31), or falls at 12 months (0.71 falls/person/year in intervention group and 0.87 falls/person/year in control group; incidence rate ratio 0.86, 95% CI 0.65 to 1.14). The intervention had a positive significant effect on device-measured daily steps and MVPA, and self-reported hours per week of walking, well-being, quality of life, and disability. No significant between-group differences were identified in other secondary outcomes.

**Conclusion:**

A physical activity and fall prevention programme including fall risk assessment and prevention advice, plus telephone-based health coaching, did not lead to significant differences in physical activity counts per minute or falls rate at 12 months. However, this programme improved other physical activity measures (ie, daily steps, MVPA, hours per week of walking), overall well-being, quality of life and disability.

**Trial registration number:**

ACTRN12615001190594.

**WHAT IS ALREADY KNOWN ON THIS TOPIC**

- Falls are an important public health problem, and older adults are at the highest risk of serious fall-related consequences.
- High-certainty evidence shows that structured exercise can reduce the rate of falls by approximately 23% in community-dwelling people aged 60+ years, but the impact of unstructured physical activity is still unknown.
- Interventions that promote physical activity to older people should consider fall prevention.
- There is a lack of large randomised controlled trials investigating the effect of combined physical activity and fall prevention interventions among older people.

**WHAT THIS STUDY ADDS**

- This study is a rigorously designed and implemented cluster randomised controlled trial to establish the effect of an individualised programme combining promotion of overall physical activity and fall prevention among people aged 60 years and over on physical activity counts per minute and falls rate.
- Over 12 months, the combined physical activity and fall prevention programme did not lead to significant differences in the co-primary outcomes of physical activity (ie, counts per minute) or falls rate.
- We found significant benefits in other measures of physical activity (ie, daily steps, moderate-to-vigorous physical activity (MVPA), hours per week of walking), well-being, quality of life and disability.

**HOW THIS STUDY MIGHT AFFECT RESEARCH, PRACTICE OR POLICY**

- Identifying interventions that promote physical activity while considering fall risk is warranted to enhance safety.
- The combined intervention was well accepted and while it did not increase physical activity counts per minute, it demonstrated improvement in other measures of physical activity (ie, daily steps, MVPA, hours per week of walking) without increasing fall risk.
- Further large trials are needed to investigate the effect of this combined intervention on physical activity and falls targeting people at high risk of falls, as well as the optimal dose of the intervention.

## Introduction

Falls are an important public health problem projected to increase in significance as the global population ages. Globally, it is estimated that over 37 million severe falls occur each year across all ages, resulting in substantial loss of more than 17 million disability-adjusted life-years and over 684 000 deaths.^1^ Falls impose a significant burden on the health system, totalling more than $50 billion in medical costs.^2^ Older people are at the highest risk of serious fall-related consequences such as hip fracture and traumatic brain injury, hospitalisation and fall-related death.^1 3^ Since many falls can lead to substantial morbidity and mortality, strategies to reverse this trend and reduce associated costs to society are needed.

Many falls are preventable and strong evidence shows that structured exercise can prevent falls, especially programmes involving a high dose of balance training.^4^ The 2020 WHO Physical Activity and Sedentary Behaviour Guidelines also recommend that older adults undertake physical activity regularly, including multicomponent exercise to enhance strength and balance and prevent falls.^5^ Although compelling evidence shows the impact of structured exercise (in particular, balance training) on fall prevention, the role of general physical activity in preventing falls remains unclear.

Conflicting associations between physical activity and falls have been reported in cohort studies.^6–9^ Also, previous trials^10 11^ observed that increases in general physical activity (specifically brisk walking) were associated with a higher incidence of falls, possibly due to the increased exposure to fall hazards in the environment. Higher levels of leisure-time physical activity were associated with falls in previous studies,^8 11 12^ and a similar association was observed in a brisk walking intervention.^10^ Taken together, this evidence indicates that interventions that promote physical activity to older people should consider fall prevention strategies.

The Coaching for Healthy AGEing (CHAnGE) intervention involved telephone-based health coaching, activity trackers and fall risk assessment and prevention advice. Our previous systematic reviews demonstrated the effectiveness of telephone-based health coaching and activity tracker interventions for increasing physical activity levels among individuals aged 60 years and older. Based on these results, we developed and pilot-tested the CHAnGE intervention among community-dwelling people aged 60+ years in a feasibility randomised controlled trial.^13^ Our results showed that such intervention was acceptable and perceived as a positive and effective approach for improving participants’ physical activity levels. However, despite the positive self-reported benefits of our intervention, this pilot study was underpowered to detect a significant effect on physical activity and falls, providing the evidence and justification to conduct a larger definitive trial.

This cluster randomised controlled trial primarily aimed to test the effectiveness of the CHAnGE intervention on physical activity and fall rates compared with a healthy eating intervention. We hypothesised that the CHAnGE intervention would lead to an increase in physical activity (ie, activity counts per minute) and a reduced fall rate compared with the healthy eating control.

## Methods

### Study design

The CHAnGE trial was a pragmatic, assessor-blinded, cluster randomised controlled trial with two parallel groups, and each community-based group recruited was considered as a cluster. The study is reported according to the Consolidated Standards Of Reporting Trials (CONSORT) statement for cluster randomised controlled trials^14^ and with reference to the Template for Intervention Description and Replication checklist.^15^ The full trial protocol and statistical analysis plan are published.^16 17^ A process evaluation conducted alongside the trial to assist with interpretation of trial outcomes and mechanisms, and to aid implementation into practice, has been previously published.^18 19^

### Participants and recruitment

We recruited community-living people from metropolitan Sydney and the regional Orange community (New South Wales (NSW), Australia) via direct contact with established community-based organisations for older people using the criteria presented in table 1.

**Table 1:** Eligibility criteria

Inclusion criteria	Exclusion criteria
Community-based organisations including members predominantly aged 60+ years and held meetings or events at least once every 2 months. Group members were potentially eligible for the trial if they: were 60+ years. were living in a private dwelling or retirement village. regularly attended meetings (at least once every 2 months) or other activities at the participating community group.	People were excluded from participation if they: self-reported undertaking 30 min of moderate-to-vigorous-intensity physical activity at least 5 days per week. had a fall risk assessment and intervention programme in the past year. had a diagnosis of dementia or a cognitive impairment assessed by Memory Impairment Screen^51^ (score <5). had insufficient English language skills to fully participate in the programme. had a progressive neurological disease. had a medical condition precluding exercise participation. were unable to leave the house without physical assistance from another person.

### Randomisation and blinding

Groups (or clusters) were randomised to either (1) the physical activity and fall prevention intervention, or (2) the healthy eating intervention. Allocation concealment was ensured by using an automatic phone-based randomisation service performed via an interactive voice response system. It used the method of minimisation, stratified by rurality, socioeconomic status and whether the group purpose was related to physical activity. Outcome assessors were blinded to intervention allocation at both the cluster and individual level throughout the trial, and statistical analyses were performed blinded for intervention or control group allocation.

### Intervention group

Participants in the clusters allocated to the intervention received the CHAnGE programme over 12 months consisting of an information booklet about increasing physical activity and preventing falls, and a 2-hour home visit by a research physiotherapist trained as a health coach, followed by telephone-based health coaching contacts. During the home visit, the participants (1) undertook the QuickScreen fall risk assessment,^20^ (2) jointly planned a fall prevention and physical activity plan with the health coach, (3) received an activity monitor (Fitbit or pedometer) and were encouraged to wear the activity monitor during waking hours on a daily basis during the intervention period to record their daily steps. The fall prevention advice involved recommendations on strength and balance exercises and guidance related to the results of the QuickScreen fall assessment, specifically addressing aspects such as vision, peripheral sensation and medications. Additionally, it included home safety tips to prevent falls.

The home visit was followed by telephone-based health coaching with contacts delivered by the same health coach (physiotherapist) who conducted the home visit. The telephone health coaching sessions focused on increasing physical activity and preventing falls and involved behaviour change techniques such as motivational interviewing, problem-solving, goal setting and action planning. During the telephone contact, health coaches also assisted participants to find suitable local exercise opportunities, to troubleshoot problems with the Fitbit, enquired about the circumstances of any falls experienced and discussed strategies for reducing future fall risk. The health coaches contacted participants via telephone approximately once a fortnight for the first 6 months and then monthly for the remaining 6 months of the study. Participants were offered up to 19 telephone coaching sessions, with no required minimum number of sessions. The home visit and telephone health coaching sessions were delivered by physiotherapists with experience working as health coaches and extensive training in empowerment-focused health coaching and additional training in using behaviour change science and self-determination theory to guide intervention. More details are provided in online supplemental table 1.

10.1136/bjsports-2023-107027.supp2 Supplementary data

### Control group

Participants in the clusters allocated to the control group were offered a 12-month nutrition programme with a booklet about healthy nutrition and access to telephone-based health coaching focused on healthy eating. The *Get Healthy Information and Coaching Service*, a free health coaching service from the NSW Ministry of Health, delivered the health coaching sessions. Participants were contacted approximately once a fortnight for the first 6 months, and then monthly for the remaining 6 months. Participants were offered a maximum of 19 telephone coaching sessions, and there was no required minimum number of sessions.

### Outcomes

#### Co-primary outcomes

The two co-primary outcome measures were: (1) physical activity, expressed as mean counts/min/day, assessed over a 7-day period using a hip-worn activity monitor (ActiGraph GT3X+ accelerometer), measured at 12 months post-randomisation, and (2) fall rates, recorded with monthly postal calendars over a period of 12 months.

#### Secondary outcomes

The secondary outcomes were the proportion of fallers (self-report via monthly surveys); daily steps (ActiGraph); self-reported physical activity (Incidental and Planned Exercise Questionnaire (IPEQ))^21^; the proportion of ActiGraph wear time in sedentary, light, moderate and vigorous physical activity; well-being (Composite Scale of Wellbeing (COMPAS-W))^22^; quality of life (EQ-5D-5L utility score and Visual Analogue Scale (VAS))^23^; disability (WHO Disability Assessment Schedule 2.0)^24^; physical activity-related goal attainment (Goal Attainment Scale)^25^; eating habits (eight questions from the Australian Health Survey)^26^; body mass index (BMI, self-reported); fear of falling (Falls Efficacy Scale International-Short Form)^27^; mood (Positive and Negative Affect Schedule)^28^; mobility-related confidence (Modified Gait Efficacy Scale)^29^ and risk-taking behaviour.^30^ The details of the secondary outcomes and follow-up time points are shown in online supplemental table 2.

10.1136/bjsports-2023-107027.supp3 Supplementary data

### Statistical analysis

#### Sample size

Based on our sample size calculation to detect between-group differences for our co-primary outcomes,^16^ 30 clusters per arm (a total of 60 clusters) and an average of 10 participants per cluster (n=600) were required for physical activity (between-group difference=10%, two-sided α=0.05, power=0.90, mean=23 counts/min, SD=120, dropout rate=20%, intracluster correlation (ICC)=0.01)^31^ (27) and falls (lower rate of falls in intervention group=30% (incidence rate ratio (IRR)=0.67), two-sided α=0.05, power=0.80, months to account for loss to follow-up=11).^32^ We assumed overdispersion in the negative binomial regression model to be 0.65 based on a previous trial,^32^ a control group rate of falls of 0.06/person/month over the follow-up period,^33^ as this was the rate in a study of a similar population, a design effect of 1.09 with an ICC of 0.01^34^ and withdrawal of six clusters. This sample size was also sufficient to detect between-group differences in the order of 10–15% for the secondary outcome measures.

#### Data analysis

We followed a predefined statistical plan.^16^ We assessed intervention effects by means of generalised estimating equations (GEE) models using an exchangeable correlation structure to account for correlation between individuals within the clusters and estimated SE using cluster robust. The number of falls/person-year was analysed using negative binomial regression models to estimate the difference in rates between the groups after 1 year (primary outcome). For the continuously scored primary (device-measured physical activity) and secondary outcome measures, Gaussian GEE regression adjusted for baseline values was used to assess the effect of group allocation. Log-binomial GEE regression was used to compare groups on dichotomous outcome measures (proportion of fallers, proportion meeting physical activity cut-off points). We used an intention-to-treat approach.

#### Planned subgroup analysis

Planned subgroup analyses assessed differential effects of the intervention by baseline physical activity levels and history of falls. We dichotomised the physical activity levels into two categories: low physical activity (<7000 daily steps) and high physical activity (≥7000 daily steps).^35^ We also categorised the history of falls into no falls, one fall, and two or more falls in the past year.

### Patient and public involvement

Four focus groups (n=26, 1.5 hours/group) involving a diverse group of older people were convened with consumers in the target age group to develop the information booklets for the intervention and control group. The physical activity and fall prevention booklet received positive feedback, and participants agreed that the information was relevant, with only minor formatting adjustments suggested. Regarding the nutrition booklet, most participants felt educated on the subject and believed they already knew how to eat well, suggesting a need for more age-relevant tips and advice, along with recommended formatting changes to enhance its appeal. Participants were not involved in setting the research question or the outcome measures or the study design.

### Equity, diversity and inclusion statement

The author group comprises individuals of various career stages (ranging from early career to senior), diverse cultural backgrounds (including Brazil, Scotland, Israel and Australia) and members of the LGBTQIA2S+ community. In our study, we included all eligible patients, irrespective of their sex, gender, race/ethnicity, cultural background or socioeconomic status, and included participants from urban and rural areas of NSW, Australia. We stratified the randomisation by area and socioeconomic status of the cluster.

## Results

### Participant flow and retention

**Figure 1:**
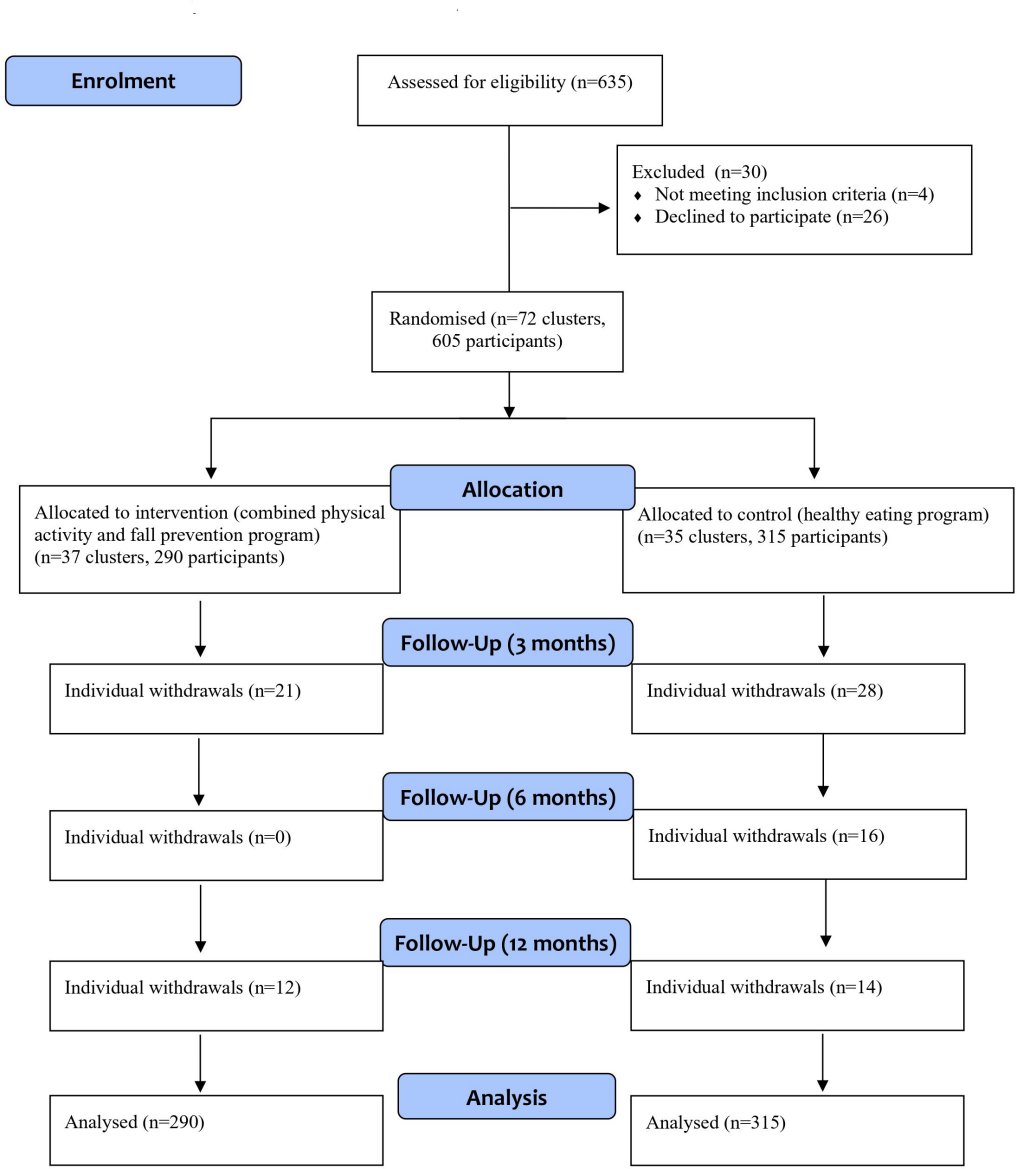
Flow of study participants (Consolidated Standards Of Reporting Trials) flow diagram.

Figure 1 presents the flow of participants through the study. Between February 2016 and September 2018, 605 participants across 72 clusters were recruited, with 37 clusters (290 participants) randomised to the intervention group and 35 clusters (315 participants) to the control group. The mean of 8.4 (SD 3.0, range 4–17) participants was recruited from each of 72 clusters. The average cluster size was smaller than originally planned (mean of 8.4 participants/cluster) and 72 clusters were recruited (n=605). Of 605 participants who were randomised, 515 (85%) and 488 (81%) completed the 6-month and 12-month device-based physical activity assessments, respectively. A total of 515 participants (85%) completed all 12 months of the falls calendars. Secondary outcomes were completed by 556 (92%), 540 (89%) and 509 (84%) participants at 3-month, 6-month and 12-month follow-ups, respectively. More participants in the control group withdrew from the study than the intervention group (control 20% (63 of 315) vs intervention 11% (33 of 290)). The main reasons for withdrawal were personal reasons (eg, competing priorities, family health issues), health related and no longer interested in the study.

### Compliance with the study protocol

Participants randomised to the intervention group received a single home visit followed by an average of 13 (SD 8, range 2–18) health coaching contacts, with each session lasting from 5 to 30 min.

### Baseline characteristics of participants

The mean age of the 605 participants was 74 (SD 8) years, 70% (n=425) were women and 30% (n=182) reported a history of falls in the past year at baseline. Participants in the two arms were similar in all respects. Table 2 presents the baseline participant characteristics.

**Table 2:** Participant characteristics at baseline (n=605)

Participant details	Intervention group (n=290)	Control group (n=315)	Total (n=605)
Age (years), mean±SD	74±7.5	75±8.5	74±8
Age (years), median (IQR)	74 (68–79)	74 (69–80)	74 (69–79)
Female, n (%)	205 (71)	220 (70)	425 (70)
Live alone, n (%)	112 (39)	108 (34)	220 (36)
Speak languages other than English at home, n (%)	9 (3)	4 (1.3)	13 (2)
Total medications (n), mean±SD	3±2.37	3±2.65	3±2.52
Total comorbidities, mean±SD	3±1.90	3±1.89	3±1.90
Use a walking aid, n (%)	5 (1.7)	6 (1.9)	11 (1.8)
Has fallen in the past year, n (%)	87 (30)	97 (31)	184 (30)
Self-rated balance fair/poor, n (%)	78 (27)	98 (31)	176 (29)
Self-rated fear of falling ≥moderate, n (%)	71 (25)	89 (28)	160 (26)
Daily steps (n), mean±SD*	6495±2976	6306±2885	6398±2929
Meeting physical activity recommendation, n (%)*†	113 (39)	125 (41)	238 (40)
Self-report physical activity (hours/week), mean±SD‡	32.1±18.7	29.1±17.2	30.51±18.0

*Accelerometer-based measure.

†>150 min of moderate-to-vigorous-intensity physical activity.

‡IPEQ-based measure.

IPEQ, Incidental and Planned Exercise Questionnaire.

### Effects of the intervention

#### Co-primary outcomes: physical activity counts and falls rate

Based on accelerometry, physical activity counts varied significantly between intervention and control groups at 6 months after randomisation (mean difference (MD) +21 counts/min/day, 95% CI 4 to 39), indicating that the intervention group significantly increased their overall physical activity compared with the control group. However, this between-group difference was not apparent at 12 months after randomisation (MD +5 counts/min/day, 95% CI −21 to 32). Group data are presented in tables 3 and 4.

**Table 3:** Mean±SD*, median (IQR) or n (%) of groups at baseline and 6-month and 12-month follow-up

Outcome measures	Groups							
	Month 0		Month 3		Month 6		Month 12	
	Intervention (n=290)	Control (n=315)	Intervention (n=269)	Control (n=287)	Intervention (n=269)	Control (n=272)	Intervention (n=257)	Control (n=252)
**CPM from accelerometer†**	267±126*	**271±126‡**	**Not assessed**	**Not assessed**	**271±146§**	**254±125¶**	262±153**	**265±168††**
Daily steps (number)†	6495±2976*	6306±2885‡	Not assessed	Not assessed	6621±3104	5847±2732¶	6445±3376**	5952±2844††
IPEQ (hours/week)								
Incidental activity	27.38±18.05	24.86±16.44	26.64±17.43	26.13±15.91	26.01±18.11	25.07±17.13	25.98±18.22	23.58±15.82
Walking activity	3.10 (1.13–6)	2.63 (1.13–5.1)	3.75 (1.88–6.93)	3.19 (1.13–5.25)	3.38 (1.69–6.75)	2.63 (0.88–5.06)	3.75 (1.69–6.75)	2.44 (1.13–4.88)
Planned activity	4.93 (1.75–8.75)	4.63 (1.75–8.13)	6.38 (3.38–10.25)	4.82 (2.63–8.38)	6 (2.69–9.63)	5.19 (2.63–8.38)	6.63 (3.38–10.25)	5.25 (3.00–8.38)
Planned walking activities	1.5 (0–3.38)	1.5 (0–3.38)	1.69 (0.75–3.38)	1.5 (0.38–3.38)	1.69 (0.75–3.38)	1.5 (0–3.38)	1.69 (0.75–3.38)	1.5 (0.25–3.38)
Planned sport activities	2.90±3.53	2.64±2.60	3.54±3.76	3.00±3.21	3.53±3.19	3.05±2.87	4.82±3.88	4.24±3.35
Total score	32.04±18.69	29.10±17.19	32.94±18.54	31.16±17.08	31.73±18.82	29.70±18.20	33.88±18.80	29.90±16.80
Device-measured sedentary, light and moderate-to-vigorous physical activity (MVPA)								
Sedentary (min/week)†	514.29±119.14*	505.6±124.21‡	Not assessed	Not assessed	519.46±138.89§	504.57±144.96¶	532.54±147.62**	504.41±157.90††
Light intensity (min/week)†	262.55±90.30*	263.01±87.15‡	Not assessed	Not assessed	255.14± 81.5§	244.17±84.49¶	255.34±78.95**	248.92±88.97††
MVPA (min/week)†	109 (39−218)*	116 (38–220)‡	Not assessed	Not assessed	120 (50−205)§	93 (32–202)¶	96 (34–209)**	93 (28−191)††
Meet recommendation of 150 min/week of MVPA, n (%)†	113 (39)	125 (41)	Not assessed	Not assessed	110 (42)	90 (35)	87 (35)	84 (35)
COMPAS-W (raw score 26–130)	98.74±11.33	98.64±10.14	100.11±10.35	99.10±10.32	100.41±10.32	98.95±10.49	93.21±7.82	92.16±8.54
EQ-5D-5L quality of life‡‡								
Health utility score (−0.68 to 1)	0.83±0.17	0.83±0.15	0.85±0.17	0.82±0.17	0.84±0.18	0.81±0.18	0.84±0.17	0.84±0.17
VAS score (0–100)	82.93±13.14	81.82±13.17	84.54±12.94	82.92±13.92	83.98±13.90	82.32±14.24	84.20±14.52	81.65±15.31
WHO Disability Assessment Schedule 2.0 (raw score 12–60)‡‡	16.47±4.76	16.83±4.64	15.73±4.05	16.16±4.78	15.99±4.70	16.64±5.11	15.58±4.17	16.29±4.83
Australian Dietary Guidelines								
Number of vegetable serves	2.68 (1.97)	2.77 (1.40)	2.71 (1.39)	3.15 (1.38)	2.82 (1.40)	3.10 (1.40)	2.78 (1.46)	3.04 (1.37)
Number of fruit serves	1.79 (1.05)	1.91 (1.13)	1.90 (0.99)	2.08 (1.17)	1.93 (1.03)	2.16 (1.31)	1.99 (1.10)	2.05 (1.02)
BMI	27.42±5.07	27.76±6.03	Not assessed	Not assessed	27.85±14.01	27.07±4.96	27.15±5.08	26.93±5.13
Falls Efficacy Scale (7−28)‡‡	9 (7−10)	8 (7−10)	8 (7−9)	8 (7−10)	8 (7−9)	8 (7−10)	8 (7−9)	8 (7−10)
PANAS Scale, (10−50)								
Positive Affect score	36.04±7.52	35.42±7.29	35.40±8.08	35.34±7.76	36.25±7.39	35.12±8.07	36.53±7.60	35.35±7.83
Negative Affect score	11 (10–15)	12 (10–14)	11 (10–15)	11 (10–14)	12 (10–14)	11 (10–14)	11 (10–14)	11 (10–14)
Modified Gait Efficacy Scale (10−100)	93 (83–98)	92 (81–98)	94 (84–98)	94 (83–98)	95 (86–99)	94 (81–98)	95 (85–99)	94 (82–98)
Risk-taking behaviour (5−20)§§	11.78±2.31	11.79±2.27	11.67±2.26	11.49±2.24	11.68±2.36	11.53±2.37	11.40±2.52	11.62±2.34

Bold row=co-primary outcome.

*n=287.

†Accelerometer-based measures.

‡n=306.

§n=260.

¶n=254.

**n=246.

††n=242.

‡‡Lower scores indicate better performance.

§§Higher scores indicate lower levels of concerning about falls.

BMI, body mass index; COMPAS-W, Composite Scale of Wellbeing; CPM, counts per minute; IPEQ, Incidental and Planned Exercise Questionnaire; PANAS, Positive and Negative Affect Schedule (Positive Affect subscale score); VAS, Visual Analogue Scale.

**Table 4:** Mean difference (95% CI; p value) or risk ratio (95% CI) between intervention and control groups

Outcome measures	Difference between groups (baseline adjusted) or risk ratio		
	Month 3 minus month 0 (n=556)	Month 6 minus month 0 (n=540)	Month 12 minus month 0 (n=509)
	Intervention minus control or intervention relative to control	Intervention minus control or intervention relative to control	Intervention minus control or intervention relative to control
**CPM from accelerometer**	**Not assessed**	21.34 (3.66 to 39.03; 0.02)*	**5.32 (−21.23 to 31.87; 0.70)**†
Daily steps	Not assessed	649 (283 to 1015; <0.001)	461 (26 to 895; 0.04)
IPEQ (hours/week)			
Incidental activity	−1.15 (−3.28 to 0.98; 0.29)	−0.71 (−3.00 to 1.59; 0.55)	0.80 (−2.05 to 3.65; 0.58)
Walking activity	0.96 (0.19 to 1.72; 0.02)	0.91 (0.18 to 1.65; 0.01)	1.13 (0.51 to 1.75; <0.001)
Planned activity	1.41 (0.45 to 2.36; 0.004)	0.88 (0.08 to 1.70;0.032)	1.33 (0.38 to 2.28; 0.006)
Planned walking activities	0.74 (0.10 to 1.38; 0.02)	0.57 (0.95 to 1.05; 0.02)	0.83 (0.28 to 1.39; 0.003)
Planned sport activities	0.55 (−0.56 to 1.16; 0.08)	0.35 (−0.20 to 0.90; 0.22)	0.56 (−0.001 to 1.11; 0.05)
Total score	0.12 (−2.03 to 2.28; 0.91)	0.16 (−2.40 to 2.73; 0.89)	2.13 (−0.55 to 4.84; 0.12)
Device-measured sedentary, light and moderate-to-vigorous physical activity (MVPA)			
Sedentary (min/week)‡	Not assessed	7.66 (−12.87 to 28.20; 0.46)	22.28 (−7.05 to 51.60; 0.14)
Light intensity (min/week)‡	Not assessed	11.91 (−1.21 to 25.04; 0.08)	7.45 (−4.36 to 19.26; 0.22)
MVPA (min/week)‡	Not assessed	29.73 (10.97 to 48.48; 0.002)	7.68 (−19.74 to 35.09; 0.56)
Meet recommendation of 150 min/week of MVPA§	Not assessed	1.21 (0.94 to 1.56; 0.14)	1.03 (0.78 to 1.36; 0.83)
COMPAS-W (raw score 26–130)‡	1.13 (−0.07 to 2.33; 0.07)	1.68 (0.38 to 2.98; 0.01)	1.25 (−0.02 to 2.52; 0.05)
EQ-5D-5L quality of life			
Health utility score (−0.68 to 1)	0.03 (0.01 to 0.05; 0.005)	0.03 (0.005 to 0.05; 0.02)	0.02 (−0.005 to 0.04; 0.12)
VAS score (0–100)	1.25 (−0.48 to 2.98; 0.16)	1.39 (−0.78 to 3.56; 0.21)	2.10 (0.34 to 3.85; 0.02)
WHO Disability Assessment Schedule 2.0 (raw score 12–60)¶	−0.26 (−0.78 to 0.27; 0.34)	−0.61 (−1.23 to 0.01; 0.05)	−0.67 (−1.29 to −0.05; 0.04)
Australian Dietary Guidelines			
Number of vegetable serves	−0.39 (−0.58 to −0.19; 0.00)	−0.23 (−0.47 to 0.002; 0.05)	−0.23 (−0.44 to −0.03; 0.03)
Number of fruit serves	−0.12 (−0.28 to 0.03; 0.11)	−0.15 (−0.31 to 0.02; 0.08)	−0.01 (−0.16 to 0.13; 0.84)
BMI	Not assessed	−0.05 (−0.36 to 0.25; 0.74)	0.25 (−0.02 to 0.53; 0.07)
Falls Efficacy Scale (7−28)¶	−0.29 (−0.67 to 0.08; 0.13)	−0.26 (−0.58 to 0.06; 0.12)	−0.36 (−0.74 to 0.03; 0.07)
PANAS Scale (10−50)			
Positive Affect score	−0.12 (−1.22 to 0.99; 0.84)	0.81 (−0.29 to 1.92; 0.15)	0.83 (−0.32 to 1.99; 0.16)
Negative Affect score	0.17 (−0.48 to 0.83; 0.60)	0.24 (−0.45 to 0.94; 0.49)	−0.05 (−0.71 to 0.60; 0.87)
Modified Gait Efficacy Scale (10−100)‡	0.15 (−1.24 to 1.55; 0.83)	1.56 (−0.04 to 3.17; 0.06)	1.28 (−0.06 to 2.61; 0.06)
Risk-taking behaviour (5−20)**	0.12 (−0.14 to 0.39; 0.36)	0.21 (−0.08 to 0.50; 0.15)	−0.15 (−0.46 to 0.15; 0.33)

Bold row=co-primary outcome.

*n=515.

†n=488.

‡Accelerometer-based measures.

§Risk ratio.

¶Lower scores indicate better performance.

**Higher scores indicate lower levels of concerning about falls.

BMI, body mass index; COMPAS-W, Composite Scale of Wellbeing; CPM, counts per minute; IPEQ, Incidental and Planned Exercise Questionnaire; PANAS, Positive and Negative Affect Schedule (Positive Affect subscale score); VAS, Visual Analogue Scale.

During the 12-month study period, 236 people (39% of participants) reported 422 falls. Of these, six participants in the intervention and nine in the control group reported falls that resulted in fractures; seven of these participants were admitted to hospital after falling (three intervention and four control). There was a 14% lower fall rate in the intervention group (193 falls, 0.71 falls/person/year, SD 1.10) compared with the control group (229 falls, 0.87 falls/person/year, SD 1.80), with the CI encompassing a 35% reduction to a 14% increase. Details of falls by intervention and control groups are presented in table 5.

**Table 5:** Fall outcome at 12 months

	Intervention	Control
Number of participants	280	304
Number of falls	193	229
Participants with at least one fall, n (%)	73 (24%)	65 (26%)
Participants with two falls, n (%)	29 (10%)	28 (9%)
Participants with three falls, (n (%)	10 (4%)	12 (4%)
Participants with four or more falls, n (%)	7 (3%)	12 (4%)
Number of injurious falls*	34	36
Mean±SD surveillance period (days)	338±84	324±95
IRR (95% CI)	0.86 (0.65 to 1.14, 0.30)	

*Injurious falls include cuts/grazes, dislocation, sprains, traumatic brain injury, fractures; IRR: Incidence rate ratio.

#### Secondary outcomes

##### Proportion of fallers

There was a slightly higher proportion of fallers in the intervention group (119 participants, 43%) than in the control group (117 participants, 39%), but this difference in proportions was not statistically significant (IRR 1.11, 95% CI 0.90 to 1.36).

##### Walking

We found that the intervention had a positive effect on daily step count: intervention participants significantly increased their daily steps more than control participants at 6 months (MD +649 steps per day, 95% CI 283 to 1015) and 12 months after randomisation (MD +461 steps per day, 95% CI 26 to 895) based on accelerometry.

Self-reported walking measures using IPEQ indicated that intervention participants significantly increased overall walking (ie, planned walking plus incidental walking) compared with the control group at 3 (MD +0.96 hours/week, 95% CI 0.19 to 1.72), 6 (MD +0.91 hours/week, 95% CI 0.18 to 1.65) and 12 months (+1.13 hours/week, 95% CI 0.51 to 1.74) after randomisation. We also found that the weekly hours spent in planned walking significantly increased in the intervention group more than control at 3 (MD +0.74 hours/week, 95% CI 0.10 to 1.38), 6 (MD +0.57 hours/week, 95% CI 0.95 to 1.05) and 12 months (MD +0.83 hours/week, 95% CI 0.28 to 1.39).

##### Device-measured sedentary, light and moderate-to-vigorous physical activity

We found no significant between-group difference in time spent in sedentary and light activities at 6 and 12 months (table 4) as measured with the ActiGraph accelerometer. Intervention participants significantly increased their time in moderate-to-vigorous physical activity (MVPA) at 6 months (MD +29 min/week, 95% CI 10.97 to 48.48) but not at 12 months (MD +8 min/week, 95% CI −19.74 to 35.09).

When dichotomised (ie, meeting vs not meeting physical activity recommendations measured by ActiGraph), a slightly higher proportion of people in the intervention group (110 participants, 42%) met MVPA recommendations (ie, undertook ≥150 min/week of MVPA) compared with control participants (90 participants, 35%) at 6 months, but this difference was not significant (risk ratio (RR) 1.21, 95% CI 0.94 to 1.56). Similar results were found in the proportion of people meeting the MVPA recommendations at 12 months (87 intervention participants, 35% vs 84 control participants, 35%; RR 1.03, 95% CI 0.78 to 1.36).

##### Well-being and quality of life

We found significant improvements in well-being (COMPAS-W) at 6 months (MD +1.68, 95% CI 0.38 to 2.98) but not at 3 (MD +1.13, 95% CI −0.07 to 2.33) and 12 months (MD +1.25, 95% CI −0.02 to 2.52). We identified a small, significant improvement of 0.03 (95% CI 0.01 to 0.05) on the EQ-5D-5L utility score at 3 and of 0.03 (95% CI 0.005 to 0.05) at 6 months in the intervention group compared with control group but not at 12 months. The intervention yielded a statistically significant increase in the quality-of-life VAS (EQ-5D VAS) (MD +2.10, 95% CI 0.34 to 3.85), indicating a 2.1-point improvement (on a 100-point scale) in EQ-5D VAS measure at 12 months, but this significant increase was not observed at 3 (MD +1.25, 95% CI −0.48 to 2.98) and 6 months (MD +1.39, 95% CI −0.78 to 3.56) (tables 3 and 4).

##### Disability

The intervention group showed reduced disability (WHO Disability Assessment Schedule) compared with the control group at 12 months (MD +0.67, 95% CI 0.05 to 1.29), but not at 3 and 6 months (tables 3 and 4).

##### Goal attainment

Participants set two physical activity-related goals at baseline. When we calculated the average Goal Attainment Scale score between the two goals, the distribution of scores at 6 months and 12 months suggested better goal attainment by the intervention group at 6 (OR 1.6, 95% CI 1.20 to 2.22) and 12 months (OR 1.83, 95% CI 1.25 to 2.68). When the Goal Attainment Scale scores were dichotomised to indicate goal attainment or not, the scores differed significantly between intervention and control group participants at 6 months (RR 1.11, 95% CI 1.00 to 1.23) but not at 12 months (RR 1.02, 95% CI 0.97 to 1.08).

##### Eating habits

A high proportion of participants in the intervention and control groups (healthy eating group) were in the maintenance stage of change (74% and 67%, respectively) at baseline, with respect to their dietary habits. This means that most participants self-described eating healthily and had done so for more than 6 months. We identified a significant difference in the number of vegetable serves consumed per day at 3 months (MD −0.39 serves 95% CI −0.58 to −0.19) and 12 months (MD −0.23 serves, 95% CI −0.44 to −0.03) in favour of the control group, but not at 6 months (MD −0.23 serves, 95% CI −0.47 to 0.002). The control group also increased the number of fruit serves relative to the intervention group, but the difference between groups was not significant at 3, 6 and 12 months (tables 3 and 4).

##### Other secondary outcomes

The analysis revealed no significant between-group differences in BMI, fear of falling (Falls Efficacy Scale International), mood (Positive and Negative Affect Schedule), mobility (Modified Gait Efficacy Scale) or risk-taking behaviour at 3, 6 and 12 months (tables 3 and 4).

#### Subgroup analysis

Planned subgroup analysis was conducted in participants who did versus did not experience falls in the 12 months prior to baseline. Although it was underpowered, the exploratory analysis suggested a fall prevention effect in those who had fallen two or more times in the past year (n=66; p for interaction=0.88; online supplemental figure 2). Planned subgroup analysis also identified a significant fall prevention effect in participants who had a lower physical activity level at baseline (n=224, p for interaction=0.001; online supplemental figure 2).

10.1136/bjsports-2023-107027.supp1 Supplementary data

#### Adjusted analysis

On the recommendation of a reviewer, we added the minimisation variables into the model. We did so, even though this was not outlined in the statistical analysis plan, as it is known to prevent anti-conservative bias in SEs.^36^ In practical terms, including the minimisation variables in the model had little effect on point or interval estimates. Following the adjustments, our conclusions remained largely unchanged, with the exception that we detected a significant improvement in self-reported planned sports activity measured using the IPEQ (MD 0.63 hours/week; 95% CI 0.09 to 1.18) (online supplemental table 3).

10.1136/bjsports-2023-107027.supp4 Supplementary data

#### Adverse events

Five adverse events from the intervention group may have been associated with the physical activity programme. These included developing knee (n=1) and shoulder (n=1) pain, foot pain (n=2), and arm fracture (n=1).

#### Process evaluation

251 (87%) intervention participants completed the survey questions on their impressions of the CHAnGE Programme at 12 months. Participants perceived the intervention programme to be beneficial with an overall rating of 8 out of 10 (SD 1.63). Intervention participants rated their physical ability to take part in the programme as 8 out of 10 (SD 1.61), and most reported no barriers to participation (n=172; 69%). Most intervention participants took part in the health coaching sessions on a regular basis (n=216, 87%) and found the sessions beneficial (mean rating 8 out of 10 (SD 1.66)). Participants viewed the use of an activity tracker (Fitbit) as valuable in motivating them to be more active (mean rating 8 out of 10 (SD 1.90)) and 41% (n=101) planned to purchase their own activity tracker. Overall, the intervention was well accepted, and most participants would recommend the programme to others (n=225; 89%). We also conducted qualitative interviews with CHAnGE intervention participants, and the results showed that most of the interviewed participants reported that the intervention increased physical and embedded practices in their day-to-day lives.^18^

## Discussion

This cluster randomised controlled trial evaluated the effect of a combined physical activity and fall prevention programme on physical activity and falls in people aged 60+ years. The CHAnGE intervention did not achieve a significant difference in activity counts or falls at 12 months. However, the intervention had significant benefits for six key secondary outcomes: daily steps, overall walking hours, quality of life, well-being, disability and goal attainment. While the primary aim of our trial was to assess the CHAnGE intervention’s effectiveness in a broad sample of older community-dwelling individuals to enhance generalisability, exploratory subgroup analyses suggested a fall prevention effect in participants who had fallen two or more times in the past year and were less active at baseline.

### Interpretation of findings and comparison with other studies

These results have important implications for translating the known benefits of physical activity and exercise for older people in Australia. Similar to our results, a previous health coaching intervention for older people showed a significant improvement in physical activity counts when the intervention contact was more frequent (ie, twice-monthly sessions), but this increase reverted to baseline levels when the contacts diminished from twice monthly to monthly.^37^ Therefore, the reduced frequency of contacts in the second half of our intervention may have contributed to the 12-month physical activity counts being lower than those at 6 months. However, we observed a significant increase in daily steps and self-report time in walking at both 6 and 12 months which may be explained by the provision of an activity tracker for the full 12 months of the intervention. Recent systematic reviews showed that interventions using activity trackers effectively increased daily steps among adults and people aged 60+ years.^38–40^

Another possible explanation for the sustained effect of the intervention on walking but not physical activity counts is that many participants chose walking as a physical activity goal at baseline. It is possible that instead of increasing their total volume of physical activity, participants may have replaced their previous physical activities with walking.^41^ It is also possible that if participants were taking part in other activities such as swimming and cycling, which were common goals among the participants, this was not captured by the accelerometer, which is accurate at recording ambulatory activities.

Consistent with a previous trial^42^ investigating the effect of walking on falls, our intervention increased daily steps and time in walking but did not prevent falls. These results are also consistent with other trials^10 43^ that found walking as a single intervention does not prevent falls. Importantly, in contrast to some physical activity trials,^10 44^ our trial increased physical activity levels (and subsequent exposure to fall-risk situations) without increasing falls. We speculate that the fall prevention advice, part of the intervention, may have assisted in this regard.

The non-significant reduction in the falls rate may have been due to the baseline participants’ characteristics. We included a relatively active group, and 70% reported no history of falls at baseline. Although our exploratory subgroup analysis was underpowered (n=69), we observed a fall prevention effect in those who had fallen ≥2 times in the past year. Further investigation of the intervention impact targeting those who have a history of two or more falls in a large fully powered trial is warranted.

### Strengths and limitations of this study

This was a large, appropriately powered pragmatic cluster trial testing the effect of a combined physical activity promotion and fall prevention programme on physical activity and falls among community-dwelling people aged 60+ years. This trial followed CONSORT statement^45^ and the protocol and statistical analysis plan were prospectively published. We included a robust randomised controlled design, with randomisation at the cluster level, a fully powered sample size, relatively long intervention (ie, 12 months), device-based measurement of physical activity and recommended method to collect fall data.^46^ We also included broad inclusion criteria and recruited a wide range of seniors’ groups across a metropolitan and a regional area of NSW, Australia. To minimise the risk of bias, a concealed random allocation to groups, blinded outcome assessment and an intention-to-treat approach were used.

Our findings, however, should be interpreted in the context of several limitations. Although we used a device-based physical activity measure, it is possible that measurement reactivity^47^ (change in behaviour from awareness of being monitored) may have biased the results (ie, acted as a co-intervention in the control group). In addition, we were unable to obtain primary outcome data on 19% of participants for the physical activity outcome and 15% for the falls outcome. We relied on self-report measures for falls and some secondary questionnaire-based outcomes, which are prone to recall and response bias.^48^ As expected in physical activity trials, participant and therapist blinding were not possible; however, this might have affected participants’ behaviour in the trial and their response to subjective outcome measures.^49^ Although we recruited participants from metropolitan and regional areas of Australia, community-dwelling older people who participated in our study had no difficulties speaking English which may impact the generalisability of our results. The analysis of multiple outcomes could lead to multiplicity issues and increase the probability of a type I error, but we prespecified.^50^ Another limitation lies in the focus on assessing multiplicative interaction exclusively, with the scale of additive interaction left unexplored. It is worth emphasising that the absence of multiplicative interaction implies the presence of additive interaction if both factors affect the outcome. Therefore, while our analysis sheds light on multiplicative interactions, the equally crucial dimension of additive interactions remains unexamined in this study. Finally, measurement bias in intention-to-treat estimates and selection bias due to missing outcome data represent additional limitations. Addressing these biases effectively typically requires instrumental variable analysis and G-estimation, which can provide unbiased treatment effect estimates in cases of non-adherence, subject to specific underlying assumptions.

### Implications for practice, research and policy

Our intervention fills a gap by promoting physical activity among older people and taking fall risk into consideration. Our intervention was well accepted, improved physical activity without increasing falls and it may have the potential to be implemented at scale. Our intervention was mostly delivered on the telephone, which could also assist older people to remain physically active in remote geographical locations and during times of social isolation and pandemic-related restrictions.

### Direction for future studies

Several unanswered questions remain for future research. It is possible that a larger fall risk reduction would have been realised if it were targeted to a population at higher risk of falls. It is also uncertain whether increasing the number of health coaching contacts would sustain the effects of the intervention on physical activity and other secondary outcomes. The optimal dose of the intervention to maximise the impact on physical activity participation among older people should be investigated. It is also unclear whether our intervention represents value for money.

## Conclusions

Our findings showed that the combined physical activity and fall prevention programme did not increase physical activity counts or reduce falls at 12 months but did lead to other significant benefits. The inclusion of a fall prevention component to the physical activity intervention yielded additional benefits in reducing the rate of falls, but this reduction was not significant over the full 12-month intervention. We also observed improvements in quality of life, well-being, disability and better goal attainment. As our intervention was well accepted, it has the potential to be implemented at scale and safely increase physical activity without increasing falls in people aged 60 years and older. However, our results also suggest that the intervention needs to be sustained at a moderately intense dose to maintain impact. The impact of the intervention on people at high risk of falls as well as the optimal dose of the intervention warrants further investigation in large trials.

## Data Availability

Data are available upon reasonable request. Deidentified participant data underlying main results may be accessed by researchers who provide a methodological proposal directed to CS (cathie.sherrington@sydney.edu.au). Approval for data access will be granted on a case-by-case basis at the discretion of the principal investigator. The data will be accessible from the date of this article’s publication and will be available for a period of 5 years thereafter. The study protocol^16^ and the statistical analysis plan are available.^17^ The corresponding author (JSO) affirms that the manuscript is an honest, accurate and transparent account of the study being reported; that no important aspects of the study have been omitted; and that any discrepancies from the study as originally planned have been explained.
