# Supplementary material for: Effect of a coaching intervention to enhance physical activity and prevent falls in community-dwelling people aged 60+ years: a cluster randomised controlled trial
Source: Br J Sports Med. 2024 Jan 22;58(7):382–91. doi: 10.1136/bjsports-2023-107027 (PMC10982628; doi:10.1136/bjsports-2023-107027)
Supplement: Supplementary data [file bjsports-2023-107027supp002.pdf]

Supplementary Table 1 Intervention description using the Template for Intervention Description and Replication (TIDieR) checklist

1. Brief name	The Coaching for Healthy Ageing (CHAnGE) trial
2. Why	Falls in older people are an important public health problem. Exercise that challenges balance can prevent falls but the role of general physical activity in fall prevention is still uncertain.
3. What materials	Participants in the physical activity/fall prevention intervention received:<br>• A printed brochure containing information about fall prevention and increasing physical activity;<br>• An assessment of their fall risk factors using the QuickScreen fall risk assessment;<br>• A wearable physical activity tracker to give feedback on the amount of daily physical activity achieved.<br>Participants in the nutrition intervention received:<br>• A printed brochure containing information about healthy eating.
4. What procedures	For the physical activity/fall prevention intervention:<br>• Telephone-based health coaching was used to identify barriers and facilitators to physical activity participation, and to provide education and support to assist participants to reduce their risk of falling, and to achieve their physical activity goals.<br>For the nutrition intervention:<br>• Telephone-based health coaching was used to identify barriers and facilitators to healthy eating, and to provide education and support to assist participants to improve their dietary habits.
5. Who provided	Health coaches with tertiary qualifications as physiotherapists or exercise physiologists delivered the physical activity/fall prevention intervention.<br>Health coaches with tertiary qualifications as dieticians delivered the nutrition intervention
6. How	The fall risk assessment and tailored fall prevention and physical activity plan was delivered during one face-to-face interview for the physical activity/fall prevention intervention arm. Health coaching for both arms was delivered via telephone contact.
7. Where	The intervention was delivered to community-dwelling people in Sydney and Orange, NSW, Australia.
8. When and how much	For the physical activity/fall prevention intervention:<br>• The face-to-face assessment and interview occurred at the beginning of the intervention period and lasted for ~2 h. The telephone-based health coaching was delivered after the face-to-face assessment and interview, once every 2 weeks for ~20 min for a total duration of 6 months, and then monthly for a further 6 months.<br>For the nutrition intervention:<br>• The telephone-based health coaching started at the beginning of the intervention period and occurred once every 2 weeks for ~20 min for a total duration of 6 months, and then monthly for a further 6 months.
9. Tailoring	For the physical activity/fall prevention intervention:<br>• The fall prevention aspect of the intervention was tailored to individual need with reference to the fall risk-assessment results. The physical activity plan was tailored to participant goals, based on current physical ability and preferences.<br>For the nutrition intervention:<br>• The healthy eating plan was tailored to participant goals, current dietary habits and preferences.
