# Supplementary material for: Effect of a coaching intervention to enhance physical activity and prevent falls in community-dwelling people aged 60+ years: a cluster randomised controlled trial
Source: Br J Sports Med. 2024 Jan 22;58(7):382–91. doi: 10.1136/bjsports-2023-107027 (PMC10982628; doi:10.1136/bjsports-2023-107027)
Supplement: Supplementary data [file bjsports-2023-107027supp003.pdf]

Supplementary Table 2 Secondary outcome measures

Outcome	Measurement/tool	Follow-up (after randomisation)
Proportion of fallers	Monthly calendars	12 months
Time spent in sedentary, light, moderate and vigorous physical activity	ActiGraph	6 & 12 months
Daily steps	ActiGraph	6 & 12 months
Physical activity-related goal attainment	Goal Attainment Scale[49]	3, 6 & 12 months
Body mass index	Self-reported height and weight	3, 6 & 12 months
Eating habits	Questions from the Australian Health Survey[26]	3, 6 & 12 months
Quality of life	EQ-5D-5L[23]	3, 6 & 12 months
Fear of falling	Short form Falls Efficacy Scale International[27]	3, 6 & 12 months
Mood	Positive and Negative Affect Schedule[28]	3, 6 & 12 months
Risk-taking behaviour	5-item self-report tool[30]	3, 6 & 12 months
Well-being	26-item composite scale of wellbeing (the COMPAS-W scale)[22]	3, 6 & 12 months
Mobility-related confidence	Modified Gait Efficacy Scale[29]	3, 6 & 12 months
Self-reported physical activity	Incidental and Planned Exercise Questionnaire[21]	3, 6 & 12 months
Disability	WHO Disability Assessment Schedule II[24]	3, 6 & 12 months
