# Supplementary material for: Effect of a coaching intervention to enhance physical activity and prevent falls in community-dwelling people aged 60+ years: a cluster randomised controlled trial
Source: Br J Sports Med. 2024 Jan 22;58(7):382–91. doi: 10.1136/bjsports-2023-107027 (PMC10982628; doi:10.1136/bjsports-2023-107027)

Supplementary Figure 2 Forest plot of intervention effect on falls by history of falls and physical activity levels at baseline

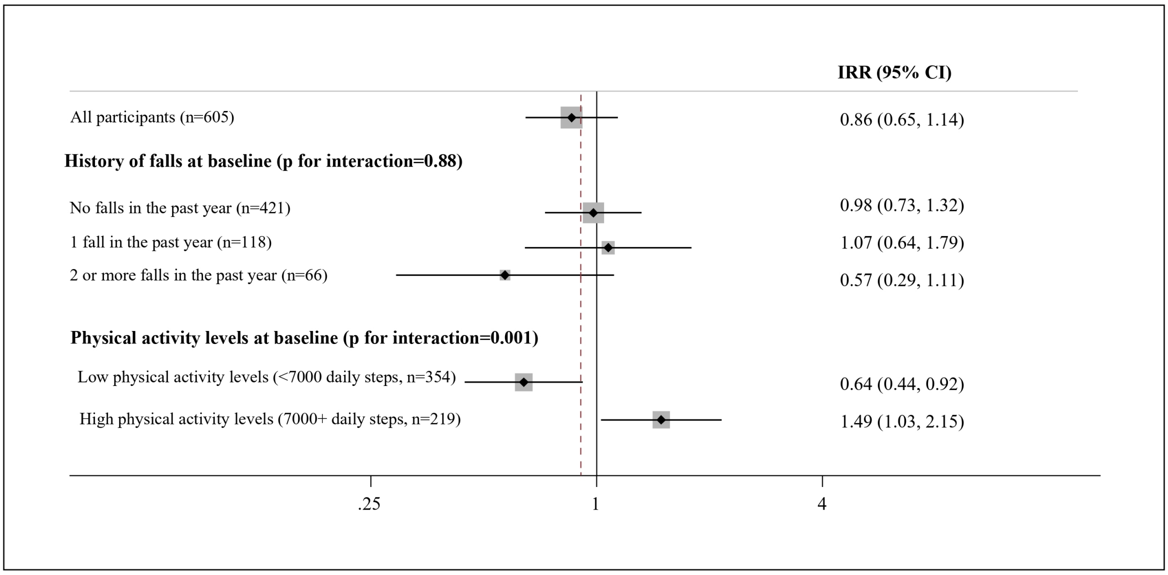

Supplement: Supplementary data [file bjsports-2023-107027supp001.pdf]
