# Supplementary material for: Effect of a coaching intervention to enhance physical activity and prevent falls in community-dwelling people aged 60+ years: a cluster randomised controlled trial
Source: Br J Sports Med. 2024 Jan 22;58(7):382–91. doi: 10.1136/bjsports-2023-107027 (PMC10982628; doi:10.1136/bjsports-2023-107027)
Supplement: Supplementary data [file bjsports-2023-107027supp004.pdf]

Supplementary Table 3 Mean difference (95% CI; p-value) or RR (95% CI) between intervention and control groups after adjusting for minimisation variables^

Outcome measures	Difference between groups (baseline adjusted) or risk ratio		
	Month 3 minus month 0 (n=556)	Month 6 minus month 0 (n=540)	Month 12 minus month 0 (n=509)
	Int minus Con or Int relative to Con	Int minus Con or Int relative to Con	Int minus Con or Int relative to Con
**CPM from accelerometer**	Not assessed	18.09 (1.35 to 34.83; 0.03)[a]	4.78 (-20.26 to 29.83; 0.70)[b]
**Daily steps**	Not assessed	558 (248 to 868; <0.001)	429 (20 to 840; 0.04)
**IPEQ (hours/week)**			
Incidental activity	-1.20 (-3.26 to 0.85; 0.25)	-0.73 (-3.01 to 1.54; 0.53)	0.87 (-2.02 to 3.76; 0.56)
Walking activity	1.02 (0.22 to 1.82; 0.01)	0.89 (0.16 to 1.63; 0.02)	1.11 (0.50 to 1.72; <0.001)
Planned activity	1.49 (0.48 to 2.50; 0.004)	0.87 (0.09 to 1.64; 0.03)	1.40 (0.47 to 2.34; 0.003)
Planned walking activities	0.79 (0.12 to 1.46; 0.02)	0.53 (0.09 to 0.98; 0.02)	0.84 (0.28 to 1.39; 0.003)
Planned sport activities	0.55 (-0.80 to 1.19; 0.09)	0.37 (-0.17 to 0.90; 0.18)	0.63 (0.09 to 1.18; 0.02)
Total score	0.16 (-1.88 to 2.18; 0.88)	0.09 (-2.43 to 2.62; 0.94)	2.30 (-0.43 to 5.02; 0.10)
**Device-measured sedentary, light, and moderate to vigorous physical activity (MVPA)**			
Sedentary (minutes/week)*	Not assessed	7.34 (-12.73 to 27.42; 0.47)	20.35 (-8.45 to 49.16; 0.16)
Light intensity (minutes/week)*	Not assessed	8.38 (-3.51 to 20.27; 0.17)	7.39 (-4.36 to 19.15; 0.22)
MVPA (minutes/week)*	Not assessed	26.66 (9.07 to 44.25; 0.003)	7.23 (-18.64 to 33.11; 0.58)
**Meet recommendation of 150 min per week of MVPA**[#]	Not assessed	1.18 (0.93 to 1.50; 0.16)	1.02 (0.80 to 1.32; 0.85)
**COMPAS-W scale (raw score 26-130)***	1.05 (-0.11 to 2.22; 0.08)	1.49 (0.21 to 2.77; 0.02)	1.14 (-0.15 to 2.43; 0.08)
**EQ-5D-5L quality of life**			
Health utility score (−0.68 to 1)	0.03 (0.008 to 0.05; 0.007)	0.03 (0.004 to 0.05; 0.02)	0.02 (-0.006 to 0.04; 0.14)
VAS score (0-100)	1.22 (-0.57 to 2.99; 0.18)	1.22 (-0.83 to 3.28; 0.24)	1.9 (0.24 to 3.54; 0.02)
**WHO Disability Assessment Schedule 2.0 (raw score 12–60)**[§]	-0.20 (-0.68 to 0.29; 0.43)	-0.48 (-1.07 to 0.09; 0.1)	-0.64 (-1.22 to -0.05; 0.04)
**Australian Dietary Guidelines**			
Number of vegetables serves	-0.39 (-0.58 to -0.19; <0.001)	-0.22 (-0.45 to 0.01; 0.06)	-0.23 (-0.44 to -0.02; 0.03)
Number of fruits serves	-0.10 (-0.25 to 0.035 0.18)	-0.15 (-0.30 to 0.01; 0.08)	-0.01 (-0.16 to 0.14; 0.89)
**BMI**	Not assessed	-0.05 (-0.36 to 0.26; 0.75)	0.25 (-0.02 to 0.53; 0.07)

Supplementary Table 3 Mean difference (95% CI; p-value) or RR (95% CI) between intervention and control groups after adjusting for minimisation variables^

Outcome measures	Difference between groups (baseline adjusted) or risk ratio		
	Month 3 minus month 0 (n=556)	Month 6 minus month 0 (n=540)	Month 12 minus month 0 (n=509)
	Int minus Con or Int relative to Con	Int minus Con or Int relative to Con	Int minus Con or Int relative to Con
**Falls efficacy scale (7-28)§**	-0.26 (-0.64 to 0.12; 0.18)	-0.25 (-0.57 to 0.07; 0.12)	-0.36 (-0.74 to 0.02; 0.06)
**PANAS scale, (10-50)**			
Positive Affect Score	-0.21 (-1.26 to 0.84; 0.70)	0.82 (-0.22 to 1.85; 0.12)	0.71 (-0.42 to 1.85; 0.22)
Negative Affect Score	0.13 (-0.51 to 0.76; 0.69)	0.27 (-0.45 to 0.98; 0.47)	-0.04 (-0.72 to 0.64; 0.91)
**Modified Gait Efficacy Scale (10-100)***	0.10 (-1.29 to 1.48; 0.89)	1.56 (-0.004 to 3.13; 0.05)	1.26 (-0.11 to 2.63; 0.07)
**Risk-taking behaviour (5-20)ǂ**	0.12 (-0.15 to 0.39; 0.38)	0.25 (-0.03 to 0.53; 0.07)	-0.11 (-0.43 to 0.20; 0.48)

Shaded row = co-primary outcome
^Minimisation variables: rural/ urban location, the cluster's socioeconomic status, and whether the cluster meeting purpose involved physical activity.

[a]n=515, [b]n=488

RR: Risk Ratio, Int=intervention group, Con= control group, CPM: Counts per minute, Incidental and Planned Exercise Questionnaire MVPA: Moderate to vigorous physical activity, BMI=Body Mass Index, PANAS: Positive and Negative Affect Schedule (Positive affect subscale score), COMPASS=composite scale of wellbeing.

*Accelerometer-based measures; #Risk ratio; §Lower scores indicate better performance; ǂHigher scores indicate lower levels of concerning about falls.
